# Examining the Environmental Ramifications of Asbestos Fiber Movement Through the Water–Soil Continuum: A Review

**DOI:** 10.3390/ijerph22040505

**Published:** 2025-03-26

**Authors:** Gergely Zoltán Macher, András Torma, Dóra Beke

**Affiliations:** 1 Department of Applied Sustainability, Albert Kázmér Mosonmagyaróvár Faculty of Agricultural and Food Sciences, Széchenyi István University, 9026 Győr, Hungary; torma@sze.hu; 2 Wittmann Antal Crop-, Animal- and Food Sciences Multidisciplinary Doctoral School, Albert Kázmér Mosonmagyaróvár Faculty of Agricultural and Food Sciences, Széchenyi István University, 9200 Mosonmagyaróvár, Hungary; 3 Department of Plant Sciences, Albert Kázmér Mosonmagyaróvár Faculty of Agricultural and Food Sciences, Széchenyi István University, 9200 Mosonmagyaróvár, Hungary; beke.dora@sze.hu

**Keywords:** asbestos, chrysotile, asbestos mobility, asbestos toxicity, soil pollution, water pollution

## Abstract

The environmental pollution potential of asbestos products is a worldwide health issue, but their dissemination through the water–soil continuum is often an overlooked aspect. Similarly, the behavior of asbestos fibers released from the products is still not fully understood, although our knowledge is based on studies concerning their mineralogical characteristics, health effects, and waste disposal. It has been claimed and contradicted that asbestos harm is only found in air and humans. Asbestos fibers are found not only in industrial settings but also through the industrial use of asbestos cement products, which has contributed to asbestos emissions and its movement in water and soil. Asbestos fibers are diverse in their physicochemical properties, and this diversity has a significant influence on their behavior in the environment. Recent research has confirmed that asbestos can be transported by water and spread to other parts of the environment. However, the mechanisms underlying this, such as the settling of fibers, their attachment to soil particles, or their movement in groundwater, as well as the environmental and health implications, require further investigation. This paper examines the process and impact of asbestos contamination in the interconnected water, soil, and plant environmental sectors, providing a systematic review of the latest literature.

## 1. Introduction

Asbestos is a naturally occurring mineral fiber with a complex and often contentious history [1,2]. While it was widely used for its heat-resistant, durable nature and insulating properties throughout the 20th century in various industrial and commercial applications [3,4], its environmental mobility remains a critical concern. Asbestos can be transported through soil and water, affecting a wide range of ecosystems and human health [5]. The widespread presence of asbestos in various environmental media, including construction materials and other industrial products, has led to significant occupational and environmental exposures [6], contributing to far-reaching health consequences. Asbestos is a collective term referring to specific naturally occurring fibrous silicate minerals [7,8]. Specifically, the term encompasses two types of silicates with a fibrous structure [9,10]: serpentines and amphiboles. Six varieties of minerals are considered fibrous asbestos. These are chrysotile, a member of the serpentine group [11], and actinolite, crocidolite, anthophyllite, grunerite, and tremolite, all members of the amphibole group [12,13]. All these minerals have different physical and chemical properties, which vary with their hazard and the manner of formation. The World Health Organization defines a regular asbestos fiber as a particle with a length greater than 5 μm, a diameter less than 3 μm [14], and an aspect ratio exceeding 3:1 [7,15]. Given their size, these minuscule fibers can easily be inhaled and can permeate through regions of the respiratory system (such as the alveoli of the lungs) [16]. When inhaled, the fibers can become lodged in lung tissue, resulting in a number of serious health problems over time [17]. Long-term exposure to asbestos has been shown to lead to asbestosis, lung cancer, and mesothelioma, a rare but aggressive cancer that develops in the lining of the lungs, chest, or abdomen [18]. Asbestos exposure is linked to serious health risks, particularly through airborne pathways, leading to strict regulations and bans in many countries. However, asbestos also remains a threat in older buildings and consumer products, where it can be transported through soil and water, posing additional environmental and public health risks. This exposure route, often overlooked, must be addressed in environmental regulations and health assessments [19,20]. Despite asbestos having been a widely utilized material, its legacy persists, posing an ongoing significant public health concern that necessitates sustained efforts to manage and remediate its presence in the environment. The widespread use of asbestos has had a significant impact on public health, with a well-established link between asbestos exposure and the development of various respiratory diseases, including asbestosis, lung cancer, and malignant mesothelioma [21,22]. Asbestos exposure can occur through both occupational and environmental sources, with construction workers, shipbuilding personnel, and individuals living in proximity to asbestos-containing materials (ACM) being at particularly high risk [23,24,25]. The health risks associated with asbestos exposure have led to the implementation of various regulatory measures aimed at mitigating its impact [26].

In the United States, the Occupational Safety and Health Administration (OSHA) has established permissible exposure limits for asbestos in the workplace [16], while the Environmental Protection Agency (EPA) regulates the removal and disposal of asbestos-containing materials. However, there are significant regulatory gaps when it comes to the presence of asbestos in soil and water, and current environmental regulations fail to address the risks associated with non-airborne asbestos exposure. These gaps highlight the need for more comprehensive environmental policies and research on asbestos contamination in these media [10,27,28].

Chrysotile, commonly referred to as white asbestos, is the most extensively utilized form of asbestos and is considered carcinogenic [29,30]. This type of asbestos exhibits distinct characteristics compared to other asbestos varieties, with a structural composition comprising octahedral magnesium and tetrahedral silicon layers, and an outermost magnesium hydroxide layer [31,32,33]. In the pH range found in soil, the outer magnesium layer dissolves rapidly, followed by the dissolution of the silicon layers, which becomes the rate-limiting step in this process [31,34,35]. Chrysotile demonstrates high solubility in moderately acidic pH conditions [31,36,37] but incongruent dissolution patterns in more acidic and neutral environments [34,35]. This indicates that the rate of white asbestos dissolution is largely influenced by the acidity of the surrounding medium [35,38]. Additionally, certain asbestos minerals, including chrysotile, exhibit a net positive surface charge in water around neutral pH, attributable to their outer brucite-like layer [39]. This surface charge behavior impacts the interaction of chrysotile fibers with environmental and biological systems, influencing their mobility and bioavailability [40,41,42]. However, crucial questions remain regarding the fate of chrysotile in soil, particularly concerning its long-term stability and potential for leaching into groundwater [33,43].

The global usage of asbestos experienced a significant rise between the 1940s and 1980s until it reached its peak [44,45]. This surge in usage was driven by the material’s favorable properties and widespread industrial applications [46,47]. However, this trend shifted when reports emerged of adverse health consequences [48] related to environmental exposure to asbestos in various European countries, including the Netherlands, Poland, and Italy [49,50,51]. These reports highlighted the serious health risks posed by asbestos, particularly in occupational settings where exposure levels were high. The detrimental effects of asbestos and asbestos-containing products were not fully recognized until the latter half of the 20th century, despite earlier warnings from researchers [52,53,54]. This delayed recognition of the health hazards associated with asbestos led to widespread occupational diseases [20], including asbestosis, lung cancer, and mesothelioma. In response to mounting evidence of its carcinogenicity, the International Agency for Research on Cancer (IARC) classified all forms of asbestos as Group 1 carcinogens in 1977 [55,56]. Despite these findings, the 1980s marked the highest point of asbestos-containing product utilization in numerous nations, such as Hungary, where asbestos was extensively used in construction and manufacturing [57]. The primary risks associated with asbestos are attributed to the inhalation of its fibers [58].

Lifetime cancer risk estimates are primarily based on the concentrations of asbestos contamination and exposure [5,59,60]. Consequently, air has been the primary medium of investigation for asbestos, with the unit of concentration being the number of asbestos fibers per volume of air [8]. Monitoring airborne asbestos levels remains a critical aspect of occupational health and safety regulations aimed at minimizing exposure and protecting public health [8].

## 2. Asbestos Cement Products and Their Environmental Risks

Over 90% of the global asbestos usage has been directed towards the production of asbestos cement sheets and pipes, which continues in certain regions today [61,62]. Asbestos cement is a composite material consisting of asbestos fibers embedded within a cement matrix, comprising cement binder and water reaction products [63]. Asbestos fibers have been extensively utilized as reinforcement in cement-asbestos composites, enhancing their tensile strength and thermal resistance [64,65,66]. Asbestos cement products represent high-binder content applications, commonly in the form of flat sheets, corrugated sheets, and pipes, where asbestos accounts for up to 8–10% of the composition, combined with 90–92% cement binder [7]. As noted, the same physicochemical properties that made asbestos useful in various engineering applications are also responsible for its environmental persistence and remediation challenges [67]. For instance, asbestos is resistant to the chemical, biological, and thermal treatments commonly employed for organic pollutants [68]. For these asbestos-containing products, a well-documented alteration process is fragmentation, cracking, and spalling, which can occur due to exogenous or anthropogenic factors [69,70]. This can lead to the release of asbestos fibers into the environment, posing significant health risks [71]. Given the hazards associated with asbestos exposure, the demolition and reclamation of asbestos-containing materials from construction and demolition waste [72] has become a major concern [71]. Several studies have explored the potential for recycling and repurposing asbestos-containing waste in a safe and environmentally responsible manner [71,73,74].

According to Tóth and Weiszburg [7], higher binder content significantly reduces the likelihood of asbestos fibers being released into the air, although the rate of spalling increases when the product is damaged. This protective effect of higher binder content is crucial in mitigating airborne asbestos exposure, particularly in construction materials where wear and tear are common [75]. However, elevated asbestos concentrations in the air surrounding dilapidated sheet-roofed buildings have been found to be detrimental to human health [76]. The degradation of these materials over time leads to the release of asbestos fibers, posing significant health risks to nearby residents and workers [77,78,79]. Asbestos cement materials, commonly used in various building applications, can release hazardous asbestos fibers into surrounding environments [80]. Climatic factors, including severe weather events and increased precipitation associated with climate change, exacerbate this issue by accelerating the deterioration of asbestos-containing materials [81]. These environmental conditions, including precipitation, water movement, evaporation, erosion, ablation, and solar radiation, as well as human activities like vehicle transport, land displacement, earthmoving, and agriculture, can facilitate the mobilization and dissemination of asbestos fibers. This contamination can impact the quality of water, air, and soil, and specifically, these processes play a crucial role in the movement of asbestos fibers through the soil profile and water systems. For instance, erosion caused by rainfall or wind can dislodge asbestos fibers from the surface of contaminated materials, such as asbestos-cement products, and transport them across the land [82]. Precipitation acts as a natural transport mechanism, washing the fibers into water bodies or deeper soil layers, especially during heavy rainfalls. Water transport further enhances the dispersal, with rivers and streams acting as conduits, carrying asbestos particles to new locations [8]. Evaporation can lead to the concentration of fibers in certain areas, and solar radiation can alter the physical properties of asbestos fibers, making them more prone to dispersal [83]. Human activities, such as vehicle transportation, can stir up dust containing asbestos fibers, facilitating their dissemination in the environment. Similarly, land displacement and earthmoving associated with construction, mining, or road building can release asbestos from buried materials, causing it to become airborne or mobilized in the soil [84]. Agricultural practices, including irrigation, plowing, and fertilizer application, can disturb contaminated soil, enabling asbestos fibers to spread more extensively. Consequently, these factors contribute to the movement of asbestos fibers into the air, water, and soil, where they can contaminate water supplies, infiltrate food chains, and pose long-term health risks to humans and wildlife [85,86]. Zhang et al. [87] identify one of the key challenges with the proposed solution, namely that the removal and disposal of asbestos cement sheets is a long-term undertaking, given the widespread use of these materials in buildings and the high costs associated with their proper removal and disposal [88]. Asbestos-containing products are typically landfilled, where they are buried in soil to prevent erosion and scattering [89]. However, wear and damage during transport or storage can lead to the formation of small asbestos fibers, which have a greater potential for water transport than bulk asbestos and can also infiltrate groundwater [8].

Furthermore, the public’s improper disposal of these hazardous asbestos cement products exacerbates the issue, as such actions can result in the unchecked erosion and release of asbestos fibers into the soil [23,25,67]. Asbestos emission from building materials, particularly in older structures, is a significant concern due to the potential health risks posed by asbestos fibers [8,20]. Several studies have found that the weathering and deterioration of asbestos-containing materials, such as cement sheets and roofing felts, can lead to the release of asbestos fibers into the air [23]. The risk is particularly heightened in urban areas with a prevalence of older buildings and infrastructure, where the fibers can become airborne and increase the likelihood of exposure among the general population [90]. The natural release of asbestos from shale can also indicate the amount of asbestos released due to the aging and weathering of the shale surface [87]. The release of asbestos fibers from naturally occurring sources adds another layer of complexity to managing environmental contamination. In a study conducted by Cely-García [91] in Colombia, activity-based sampling was used to evaluate the release of asbestos fibers from various contaminated soil samples, revealing a relatively low asbestos risk. This finding suggests that, under certain conditions, the risk of asbestos fiber release from soil may be manageable [92]. However, contrasting findings from Bornemann and Hildebrandt [81] reported that older sheet roofs or those with damaged surfaces can emit asbestos fibers into the air at average annual concentrations of several grams [93]. This substantial release poses significant health risks, particularly in urban areas with older infrastructure [94]. Additionally [76], previously documented that the surface of asbestos cement sheets undergoes weathering-induced corrosion at a rate of approximately 0.01–0.024 mm per year [76,87]. This gradual degradation process continuously releases asbestos fibers into the environment, contributing to long-term contamination and health risks [20]. The ongoing weathering and deterioration of asbestos-containing materials, combined with improper disposal practices, highlight the critical need for effective asbestos management and remediation strategies to protect public health and the environment [95,96].

Asbestos fibers are known to be highly durable and can remain suspended in the air for long periods, posing a persistent threat to respiratory health [97]. The latency period for these diseases can be several decades, meaning that the health effects of asbestos exposure may not become apparent until many years after the initial exposure [98]. This underscores the importance of proactive measures to minimize asbestos release and exposure. Moreover, the challenge of managing asbestos contamination is compounded by the variability in the types of asbestos fibers and their differing toxicological properties [8,26]. Chrysotile, amosite, and crocidolite are among the most common types, each with unique characteristics and health risks [38]. Effective asbestos management requires a comprehensive understanding of these differences and the implementation of tailored strategies to address the specific risks associated with each type [99,100]. The emission of asbestos fibers from both building materials and natural sources represents a multifaceted environmental health challenge [96]. Urban areas with aging infrastructure are particularly vulnerable, and the continuous weathering of asbestos-containing materials further exacerbates the risk [20]. Studies have shown varying levels of risk associated with asbestos release from different sources, highlighting the need for ongoing research and adaptive management strategies [101]. To protect public health, it is imperative to develop and enforce stringent regulations on asbestos use, ensure proper disposal practices, and invest in the remediation of contaminated sites [26]. Public awareness campaigns and education on the dangers of asbestos exposure are also crucial components of a comprehensive asbestos management plan [26].

## 3. Soil Contamination by Asbestos Complexes and Its Risks

Asbestos, a naturally occurring mineral, can become a significant environmental pollutant when released into the soil [102]. The terrestrial environment can be contaminated with asbestos through natural [103,104,105] or human-induced processes [33]. Naturally occurring asbestos in rocks is transferred to the soil through geological phenomena [106], resulting in the soil inheriting the mineralogical and geochemical properties of the underlying bedrock [69,107]. Natural asbestos deposits are discontinuous, highly tectonized, and fragile and can break down under minimal mechanical stress [108], transforming into incoherent material that can be easily incorporated into the soil. Asbestos fibers can be easily incorporated into soil through natural weathering processes or human activities, such as the degradation of asbestos-containing construction materials [82,102,104].

Asbestos may enter soils and sediments through natural processes but primarily through release from anthropogenic sources [109,110]. Nonetheless, naturally occurring asbestos fibers in rocks and soils can also pose an environmental risk by becoming airborne. Previously, soil was considered a layer that effectively insulated asbestos in environmental waste [106,111]. Recent studies suggest that organic acids commonly found in soils can enhance the leaching of small asbestos fibers [76]. Although the transport rate of these fibers is not rapid, they can become a source of respirable dust in the air due to various mechanical factors [112,113]. Dry conditions, strong winds, and mechanical disturbances—such as excavation, construction activities, or heavy machinery movement—can further contribute to fiber resuspension, increasing the likelihood of airborne asbestos exposure [114]. Additionally, seasonal variations in soil moisture levels may influence fiber stability, with prolonged droughts exacerbating dust generation and fiber dispersal [115]. Without proper mitigation measures, these processes can lead to long-term contamination risks, affecting both agricultural workers and nearby populations.

Once present in the soil, asbestos can alter its physical, chemical, and biological properties in multiple ways, thereby influencing soil quality and ecosystem health [116]. Physically, asbestos fibers can modify soil texture and porosity, potentially affecting water retention and infiltration rates. These changes may lead to altered soil aeration, influencing root growth and microbial activity [89]. Chemically, asbestos minerals can interact with soil components, affecting pH levels and the availability of essential nutrients. Some studies suggest that asbestos-containing minerals can release magnesium, iron, and silica into the soil, which may alter nutrient cycling and plant uptake dynamics [117,118,119]. Biologically, asbestos contamination can impact microbial communities by disrupting their composition and metabolic functions. Certain fungi and bacteria capable of interacting with silicate minerals may play a role in asbestos weathering, but the overall ecological consequences remain poorly understood [120,121,122]. The mechanisms of asbestos entry into the soil profile include atmospheric deposition, industrial waste disposal, and weathering of asbestos-containing rocks. Once in the soil, fibers can adsorb onto mineral surfaces or organic matter, influencing their mobility. While asbestos is relatively immobile under neutral to alkaline conditions, acidic environments, and organic acids can facilitate fiber desorption and leaching. Additionally, surface runoff and erosion can transport asbestos particles into aquatic systems, where they may pose further environmental and health risks [100].

The mobility of asbestos fibers in soil is influenced by various factors, including fiber characteristics, soil properties, and environmental conditions [82,102,123]. Asbestos concentrations in soil can lead to partial displacement, including re-entrainment into the air, as well as changes in the electrical charge of asbestos fibers that can affect the organic soil layer and facilitate fiber migration [124]. In natural settings, asbestos can occur in large deposits, with chrysotile being the most commonly found form, as its fibers are present in serpentine rock formations [106]. The primary anthropogenic sources are asbestos cement sheets, tiles, and panels used in construction, which predominantly contain chrysotile [67]. Environmental concentrations can vary widely due to human activities, land use, and natural factors, as the aging of asbestos cement products is the main cause of fiber release in urban environments, depending on the source type, degradation level, and treatment/manipulation [8,89,106].

The limited research on the mobility of asbestos fibers in porous media restrains our understanding of the environmental factors influencing the transport of fibers in soil [76]. The previously established relationship between fiber concentration and measured soil concentration, first demonstrated by Addison et al. [125] and expanded upon by the RIVM National Institute for Public Health and the Environment, can be leveraged to quantify asbestos levels in soil [60,112]. However, the risks of asbestos in soil remain uncertain, necessitating further research to evaluate the relationship between fiber release and health impacts [126,127]. The mobility of asbestos fibers in soil can be estimated based on studies of the transport of mineral colloids in water [128].

The distinctive physical and surface chemical characteristics of asbestos fibers, including their high aspect ratio and lengths of up to 100 μm [129], significantly influence their mobility, retention, and persistence in soil environments [67,112,130]. Unlike spherical colloids, asbestos fibers exhibit complex transport dynamics due to their elongated structure, which affects their interaction with soil particles, water flow pathways, and biological components. According to [89], the transport and removal of colloids in soil are influenced by both physical and chemical factors. Among the physical factors, particle size [131], shape [132], and soil pore size distribution [133] determine the extent to which asbestos fibers migrate or become immobilized. Larger fibers, particularly those exceeding the typical pore sizes in fine-textured soils such as clay and silt, are more likely to be trapped by mechanical filtration or straining. Conversely, smaller fibers may be transported more easily through macropores, preferential flow pathways, and fractures in structured soils. The fibrous nature of asbestos also allows for entanglement with organic matter and root systems, which can further restrict mobility.

Chemical factors significantly modulate asbestos fiber stability and movement within the soil matrix. The pH of the soil [134] plays a crucial role in determining the surface charge of asbestos minerals, which in turn affects their adhesion to soil particles. Under acidic conditions, the positive surface charge of asbestos fibers may enhance their interaction with negatively charged clay minerals and organic matter, leading to increased retention. In contrast, alkaline conditions can promote dispersion, enhancing mobility in groundwater and surface runoff. The ionic strength [135] of the soil solution further influences fiber behavior. High ionic strength environments, often associated with saline or contaminated soils, can lead to particle aggregation and flocculation, reducing the likelihood of fiber transport. Conversely, lower ionic strength may promote fiber dispersion, increasing the potential for groundwater contamination. The presence of phosphates and other solutes [136] can also impact asbestos mobility by altering electrostatic interactions, either stabilizing fibers in suspension or enhancing their adsorption onto mineral surfaces [113]. In addition to these factors, soil organic matter content plays a dual role in asbestos transport. Organic compounds, including humic and fulvic acids [137], can bind to asbestos surfaces, enhancing their retention in the soil profile. However, under certain conditions, organic ligands may also facilitate fiber mobilization by altering surface charge characteristics and promoting desorption [138]. Furthermore, microbial activity within the soil can influence asbestos weathering processes, potentially leading to fiber fragmentation or dissolution, which may modify their environmental impact [89]. Colloids are transported through the soil by infiltrating water and deposited on soil particles through sedimentation, absorption/adsorption, and diffusion, as predicted by colloid filtration theory [89,139]. Previous research on asbestos in soils has primarily focused on former asbestos mining areas [113], but there is also a need to assess other impacted regions and determine their level of exposure.

Moreover, agricultural activities can cause asbestos particles in the soil to decompose and become airborne [113,140,141], rendering the agricultural sector particularly vulnerable despite the paucity of studies in this domain [89,125,142]. The disturbance of soil through plowing, irrigation, and other farming practices can enhance the risk of asbestos fiber release, posing health risks to farmers and surrounding communities [8]. Additionally, irrigation can facilitate fiber transport through water movement, while plowing and soil tillage may increase fiber resuspension and bioavailability [143]. Fertilizer application, especially when combined with organic amendments, could further influence fiber interactions in the soil matrix, potentially altering their persistence and uptake by crops [144].

Additionally, remediation of asbestos-contaminated soils presents unique challenges. Traditional soil remediation techniques may not be effective for asbestos fibers due to their size, durability, and tendency to become airborne [67]. Innovative approaches such as phytoremediation, where certain plants are used to stabilize and contain asbestos fibers, and in situ stabilization, where chemical agents are added to soil to bind fibers and reduce mobility, are being explored [145]. These methods aim to mitigate the release of asbestos fibers and reduce the associated health risks [120].

The presence of asbestos in soil is a complex issue with significant implications for environmental and public health [106]. The mobility of asbestos fibers in soil, influenced by various physical and chemical factors, underscores the need for comprehensive research and targeted remediation strategies [89]. Understanding the interaction of asbestos fibers with soil and developing effective management practices are crucial steps in mitigating the risks associated with asbestos contamination [26]. Further studies are essential to evaluate the long-term impact of asbestos in soil and to develop innovative solutions to protect human health and the environment [100].

## 4. Water Contamination by Asbestos Complexes and Its Risks

In stark contrast, the potential dangers of waterborne exposure to ingested asbestos have received considerably less scrutiny despite studies indicating an elevated risk of stomach cancer from such exposure [146]. This discrepancy in research focus is notable, given the substantial health implications. Asbestos-containing materials primarily pose a hazard when their fibers are released into the air, creating an inhalation risk. However, the pathogenicity of ingested asbestos fibers remains a critical and ongoing area of investigation. Studies have explored various aspects of this issue, including early examinations by [147,148,149] and more recent work by [150]. Despite these efforts, the body of literature remains less extensive compared to airborne asbestos risks [57]. Furthermore, very few studies have delved into the potential risks arising from airborne asbestos and asbestos-forming minerals that are released during water mobilization or evaporation processes [151]. This gap in research is significant, as it suggests that there could be overlooked pathways for asbestos exposure [152]. Previous research had suggested that environmental exposure to asbestos fibers through water or soil was negligible because the fibers were assumed to adhere to soil particles and be filtered out during infiltration [153]. However, this assumption has been challenged by more recent studies reporting the presence of asbestos fibers in deep underground aquifers [105,154,155]. These findings indicate the emergence of an alternative transport pathway for asbestos exposure through shallow groundwater systems [5,89,156].

The transport of asbestos fibers from soil to surface water is highly dependent on a range of site-specific factors, including precipitation patterns, soil and rock degradability, slope morphology, vegetation cover, and the degree of anthropogenic influence [157]. This complex interplay of factors means that understanding the environmental conditions that enhance the mobility of asbestos fibers between contaminated soil and groundwater is of utmost importance [89]. This knowledge is crucial for developing effective strategies to mitigate the health risks associated with both airborne and waterborne asbestos exposure, ensuring better public health outcomes in areas affected by asbestos contamination [26]. Asbestos fibers can infiltrate groundwater sources through the natural weathering of asbestos-containing rocks and improper disposal of asbestos-containing waste [158]. Previous research [157] has concentrated primarily on surface water contamination by asbestos, while groundwater studies remain in the experimental phase. In general, asbestos pollution in water is attributable to both anthropogenic and natural factors [8]. Studies have shown that chrysotile asbestos can be present in groundwater near mining sites, contributing to potential health risks. The persistence of asbestos fibers in groundwater is largely due to their resistance to chemical and biological degradation [159]. Groundwater contamination by asbestos is a concern near industrial sites where asbestos was historically used or disposed of. Asbestos fibers in groundwater can be transported over long distances, posing risks to distant populations [8,120].

Of particular note is the fact that the impact of asbestos fibers on aquatic ecosystems is largely unknown, and very few studies have been conducted to elucidate the various mechanisms of action [160]. In countries where industrial asbestos use is prohibited or strictly regulated, anthropogenic sources include the disposal of asbestos-contaminated water from mines and quarries [8]. Another anthropogenic source of emissions is the erosion of asbestos cement pipes used in drinking water and wastewater networks, which can also release asbestos fibers [161]. Additionally, water flowing through improperly disposed asbestos-contaminated waste can become a source of pollution [89]. Although extensive research has been conducted on the health hazards [162] associated with the inhalation of airborne asbestos and analogous asbestos-like minerals [151], there remains a paucity of information regarding the potential health risks posed by asbestos fibers suspended in water, which represents one of the most concerning mobilization pathways in the environment [25,163]. Caramuscio et al. [164] investigated the presence of asbestos contamination in groundwater and surface water near an asbestos mine. Their findings revealed that the asbestos content of stream water near the mine ranged from 1.00 to 3.60 mg/L, while groundwater levels were between 1.00 and 4.10 mg/L. Previous studies [155,165,166], as reported [167], have demonstrated that asbestos pollutants from mining activities can lead to metal contamination and increased asbestos levels in surface waters and sediments [168,169,170], particularly in areas surrounding active and inactive asbestos mines [155,171,172]. The asbestos fibers detected were predominantly short, measuring less than 5 µm in length, but the potential for asbestos dispersion from stream water to air was not examined [83,173,174]. Despite the recognized health risks, European Union regulations on water quality still do not specify limit values or guideline values for asbestos in drinking water [175,176], nor for natural surface and groundwater. This regulatory gap presents significant challenges in assessing and mitigating exposure risks, particularly in regions where aging asbestos cement pipelines, industrial effluents, or natural geological sources contribute to fiber release [8,177].

The absence of harmonized standards for asbestos monitoring in water bodies impedes the development of risk-based management strategies and hinders cross-border comparability of exposure data. Moreover, the lack of legally binding thresholds limits enforcement mechanisms, potentially delaying preventive interventions even in areas with documented contamination. Given the growing body of evidence on the potential carcinogenicity of ingested asbestos fibers, the absence of EU-wide regulations may result in insufficient protection of public health, particularly for vulnerable populations relying on untreated or minimally treated water sources [178]. Without targeted epidemiological studies and toxicological assessments, the long-term implications of chronic low-dose asbestos ingestion remain uncertain, reinforcing the need for regulatory advancements and precautionary policy measures at both national and supranational levels [179,180].

The World Health Organization (WHO) has yet to establish a safe concentration level for asbestos in water, a notable omission given the organization’s global influence on health standards [181]. In contrast, the United States Environmental Protection Agency (US-EPA) has set a maximum contaminant level for asbestos in drinking water at 7 × 10^6^ asbestos fibers per liter, primarily addressing fibers longer than 10 µm [182,183]. This standard reflects a more proactive approach to managing the risks associated with asbestos in water. Several studies have focused on monitoring and determining the background levels of asbestos in drinking water, with some of the highest values measured exceeding 10^7^ asbestos fibers per liter in the USA [184,185,186] and 10^8^ asbestos fibers per liter in Canada [187]. These findings underscore the variability and potential for high concentrations of asbestos in water sources. Further complicating the issue [186] demonstrated a correlation between airborne and waterborne asbestos in buildings where asbestos [chrysotile and amphibole] was detected in tap water [8]. They reported that 2.4 × 10^7^ asbestos fibers per liter of water could result in air concentrations as high as 120 asbestos fibers per liter. This correlation suggests that asbestos fibers in water can become airborne, thereby increasing the risk of inhalation and the associated health impacts [151]. Consequently, potential emergency situations should not be overlooked, as the concentration of asbestos in surface waters and drinking water can dramatically increase during natural disasters [188]. Natural disasters can disrupt and mobilize asbestos fibers, leading to sudden spikes in contamination levels that pose immediate health risks [189]. From an agricultural and water management perspective, special attention should be given to the use of contaminated groundwater for irrigation [113] and indoor water usage [190]. Using contaminated water for irrigation can introduce asbestos fibers into the food chain, while indoor water use can increase the risk of asbestos inhalation as fibers become aerosolized during household activities [89]. Despite these concerns, there is a lack of research on the extent to which asbestos fibers bioaccumulate in plants and how they are transferred through trophic levels. The mechanisms of fiber uptake by plant roots, potential translocation within plant tissues, and the implications for human and animal consumption remain largely unexplored. Additionally, the physicochemical properties of asbestos may influence its interaction with soil microbiota and plant exudates, potentially altering bioavailability and accumulation patterns [42,121]. These knowledge gaps highlight the urgent need for targeted studies on asbestos behavior in agricultural systems to better inform risk assessments and regulatory frameworks. These scenarios elevate human exposure levels and underscore the need for comprehensive monitoring and regulation to mitigate the risks associated with asbestos in water. Addressing these issues requires coordinated efforts at the policy, research, and community levels to ensure that water quality standards are robust and protective of public health [191,192].

## 5. Conclusions

In our research, we have meticulously elucidated that the issues stemming from asbestos and various asbestos-containing products have long constituted a critical environmental domain, one that fundamentally transcends the traditional boundaries of waste management concerns. This study underscores the importance of re-evaluating our understanding of asbestos-related environmental hazards, particularly in light of recent developments in international scholarly discourse. The prevailing assumption that asbestos fibers remain immobile in soil and that water, as a transmission medium, has only a minimal role in the mobilization of these fibers has been robustly challenged by contemporary research findings. Table 1 presents a comprehensive critical analysis of the impacts on soil and water, as well as the identified gaps in the current body of research. These findings suggest a significantly higher potential for asbestos fiber dispersion, thereby elevating the risks of human exposure and associated health hazards. Despite the ongoing experimental nature of research in this field, a significant gap persists in the availability of standardized analytical methods and procedures. These methods are crucial for providing concrete guidelines for assessing the contamination levels in various water and soil types. The absence of such standardized protocols hampers the ability to accurately quantify and address the extent of asbestos contamination in different environmental media.

Previous investigations have typically reported very low concentrations of asbestos, predominantly consisting of fibrous chrysotile asbestos.

However, these findings should not be misconstrued as indicative of a negligible risk. Rather, they highlight the necessity for more nuanced and comprehensive analytical approaches. Furthermore, the methodology for assessing water contamination due to asbestos remains rudimentary and parallels the approaches used for air quality assessments. This underscores the urgent need for developing more sophisticated procedures that can accurately quantify asbestos contamination in water bodies on a volumetric basis. The current reliance on air-based assessment models for water contamination is inadequate and does not capture the complexity of asbestos dispersion in aqueous environments.

Given these insights, it becomes evident that future research and policy frameworks must adopt a holistic approach to addressing the asbestos problem. This involves a thorough and integrated examination of air, soil, and water as interconnected environmental spheres and transmission media. Such a comprehensive approach will enable a more accurate assessment of the environmental and health risks posed by asbestos, thereby facilitating the development of more effective mitigation and management strategies. In conclusion, our research advocates for a paradigm shift in the way asbestos-related environmental hazards are understood and addressed, emphasizing the need for integrated, multidisciplinary investigations and interventions.

## Figures and Tables

**Table 1:** Impacts on the water–soil system and associated research gaps.

Aspect	Effects on Water	Effects on Soil	Gaps and Research Needs
Physical characteristics	Asbestos fibers can remain suspended in water and settle slowly [8].	Persist in soil for extended periods without natural degradation [82].	Lack of precise data on sedimentation and transport dynamics.
Chemical stability	Type-dependent, but generally does not degrade in water [83].	Stable structure, slow weathering [193].	Limited data on long-term transformations and interactions.
Contaminant adsorption	Can adsorb heavy metals and organic pollutants [194].	May bind toxic substances, making them bioavailable to plants [195].	Unclear which contaminants can accumulate.
Biological effects	Not a food source but can enter the food chain [86].	Potentially toxic to microorganisms and plants [196].	Insufficient research on ecosystem-level impacts.
Drinking water contamination	Asbestos fibers can be present in drinking water [8].	Can leach from soil into groundwater [8].	Unclear relationship between exposure and health risks.
Toxicity	Potentially carcinogenic if inhaled [197].	Long-term presence may pose risks to living organisms [198].	Further studies needed on effects on soil-dwelling organisms.
Degradation and removal	Does not degrade naturally, only removable through physical filtration [199].	Remains in soil unless mechanically disturbed [119].	Development of effective removal techniques required.
Entry into surface waters	Introduced via rainfall and industrial discharge [67].	Transported by wind erosion and precipitation [200].	Transport mechanisms are not fully understood.
Ecological impact	May clog filter-feeding aquatic organisms [199].	Can alter soil microbial activity and reduce biodiversity [113].	Limited experimental data on ecological damage.
Sedimentation rate	Slow sedimentation, but turbulence can enhance dispersion [151].	Movement varies across different soil types [200].	Poorly understood mobility in various soil compositions.
Water treatment challenges	Difficult to remove with standard filtration methods [201].	Soil remediation is complex and costly [119].	Need for innovative methods for effective removal.
Groundwater contamination	Can infiltrate deeper layers via precipitation [151].	Mobility depends on soil pH and organic matter content [67].	Limited data on movement and concentrations in groundwater.
Temperature effects	Temperature variations may influence sedimentation [201].	Freeze-thaw cycles could alter distribution [202].	Insufficient knowledge of the impact of extreme climate conditions.
Impact on plants	Can be absorbed by plants through water uptake [198].	Toxic effects on root systems and plant growth [198].	Limited research on cellular effects in plants.
Interactions with sediments	May accumulate in sediments [67].	Functions as a temporary reservoir, releasing fibers back into the environment over time [20].	Further research needed to understand storage and mobilization in sediments.
Hydrodynamic influence	Flow conditions affect distribution [199].	Behavior varies in soils with different porosity levels [67].	Limited modeling data on flow velocity impacts.
Human exposure	Inhalation or ingestion possible through drinking and bathing [8].	Can be inhaled or ingested as soil dust [113].	Need for epidemiological studies to assess exposure risks.
Long-term environmental impact	May persist in aquatic environments for decades [199].	Can remain unchanged in soil for centuries [67].	Lack of long-term data on degradation or transformation potential.
